# Learning from biomedical linked data to suggest valid pharmacogenes

**DOI:** 10.1186/s13326-017-0125-1

**Published:** 2017-04-20

**Authors:** Kevin Dalleau, Yassine Marzougui, Sébastien Da Silva, Patrice Ringot, Ndeye Coumba Ndiaye, Adrien Coulet

**Affiliations:** 1 LORIA (CNRS, Inria Nancy-Grand Est, University of Lorraine), Campus Scientifique, Nancy, France; 2 Ecole nationale supérieure des mines de Nancy, Campus Artem, Nancy, France; 3 UMR U1122 IGE-PCV (INSERM, University of Lorraine), 30 Rue Lionnois, Nancy, France

**Keywords:** Linked data, Pharmacogenomics, Data mining, Knowledge discovery from databases, Machine learning, Valid pharmacogenes

## Abstract

**Background:**

A standard task in pharmacogenomics research is identifying genes that may be involved in drug response variability, i.e., pharmacogenes. Because genomic experiments tended to generate many false positives, computational approaches based on the use of background knowledge have been proposed. Until now, only molecular networks or the biomedical literature were used, whereas many other resources are available.

**Method:**

We propose here to consume a diverse and larger set of resources using linked data related either to genes, drugs or diseases. One of the advantages of linked data is that they are built on a standard framework that facilitates the joint use of various sources, and thus facilitates considering features of various origins. We propose a selection and linkage of data sources relevant to pharmacogenomics, including for example DisGeNET and Clinvar. We use machine learning to identify and prioritize pharmacogenes that are the most probably valid, considering the selected linked data. This identification relies on the classification of gene–drug pairs as either pharmacogenomically associated or not and was experimented with two machine learning methods –random forest and graph kernel–, which results are compared in this article.

**Results:**

We assembled a set of linked data relative to pharmacogenomics, of 2,610,793 triples, coming from six distinct resources. Learning from these data, random forest enables identifying valid pharmacogenes with a F-measure of 0.73, on a 10 folds cross-validation, whereas graph kernel achieves a F-measure of 0.81. A list of top candidates proposed by both approaches is provided and their obtention is discussed.

**Electronic supplementary material:**

The online version of this article (doi:10.1186/s13326-017-0125-1) contains supplementary material, which is available to authorized users.

## Background

Pharmacogenomics (PGx) studies how individual gene variations cause variability in drug responses [1]. Well established knowledge in PGx constitutes a basis for implementing personalized medicine, i.e., a medicine tailored to each patient by considering in particular her/his genomic context. The state of the art of this domain lies both in the biomedical literature and in specialized databases [2, 3], but a large part of it is controversial, and not yet applicable to medicine. Indeed, this results from studies difficult to reproduce and that do not fulfill statistical validation standards for two main reasons: the small size of populations involved in studies because of the rarity of gene variants studied and the potential coaction of several variants [4, 5]. It is consequently of interest to the PGx community to explore any source of evidence that may contribute to confirming or moderating PGx state of the art. So far, existing works used either molecular network databases or the biomedical literature (see “Discovery of pharmacogenes” subsection). We propose in this work to explore how other resources, and particularly Linked Open Data (LOD) may be useful in this domain.

### Linked open data

LOD are constituting a large and growing collection of datasets that present the main advantages of being represented in a standard format (based on both RDF and URIs) and partially connected to each other and to domain knowledge represented within semantic web ontologies [6]. For these reasons, LOD offer novel opportunities for the development of successful data integration and knowledge discovery campaign, as required for the discovery of novel pharmacogenes. LOD are part of a community effort to build a semantic web, where web and data resources can be interpreted both by humans and machines. The recent availability of LOD is particularly beneficial to the life sciences, where relevant data are spread over various data sources with no agreement on a unique representation of biological entities [7]. Consequently, data integration is an initial challenge one faces if one wants to mine life science data considering several data sources. Various initiatives such as Bio2RDF, the EBI platform, PDBj and Linked Open Drug Data (LODD) aim at pushing life sciences data into the LOD cloud with the idea of facilitating their integration [8–11]. It results from these initiatives a large collection of life-science data, unequally connected but in a standard format and available for mining. Despite good will and emerging standard practices for publishing data as LOD, several drawbacks make their use still challenging [12, 13]. Among existing difficulties we can cite the limited amount of links between datasets and the limits of implementations of federated queries.

### Pharmacogenomics data and linked data

PharmGKB is a comprehensive database about PGx that includes manually annotated gene–drug relationships [3]. Recently, annotations of PharmGKB have been completed with a *level of evidence* going from 1 to 4, distinguishing well validated gene–drug relationships (level =1−2) from insufficiently validated ones (3–4), thus pointing at knowledge in need for additional investigations [14]. PharmGKB does not provide its data in RDF, but parts of PharmGKB have been transformed and published in RDF by contributors of the Bio2RDF project, thus enabling SPARQL queries [15]. Clinical annotations of PharmGKB are however not freely available. Their usage is granted through a license agreement, preventing the data from being redistributed, thus published as Linked Open Data. Many other databases provides data that are indirectly relevant to PGx. For instances, DrugBank [16] provides drug–target relationships; ClinVar [17] provides gene variant–phenotype relationships; SIDER [18, 19] and Medi-Span provides drug–phenotype relationships such as drug adverse events or indications [20]. Medi-Span is a proprietary database of Wolters Kluwer Health (Indianapolis, IN) aiming at providing drug clinical data to clinicians. DGIdb (The Drug Gene Interaction database) is another interesting initiative that integrates quasi-exhaustively data about gene–drug relationships, considering 15 distinct sources [21]. DisGenet is a data integration initiative that focuses on gene–disease relationships and provides data in RDF, including parts of ClinVar and OMIM [22].

Data integration effort clearly oriented to PGx applications are less common, particularly if considering semantic web approaches [23]. Hoehndorf et al. integrated and made available a set of PGx related data that includes PharmGKB, DrugBank and CTD (the Comparative Toxicogenomics Database), using semantic web technologies [24]. They used the integrated dataset to identify pathways that may be perturbed in PGx. In this effort of publishing PGx data, Coulet et al. extracted about 40,000 PGx relationships from the biomedical literature and published them in the form of RDF statements [25].

### Mining linked data

Suggesting valid pharmacogenes in this work is seen as proposing novel gene–drug relationships from an RDF graph, which in turn can be described as a link prediction problem. Many works have focused on the link prediction problem, studying various approaches such as machine learning [26, 27], graph mining [28–30], identity resolution [31, 32] and data visualisation [33]. Some of these methods obtain good results, but all are dependent from the input graphs (its quality, topology, etc.) and are hard to reuse for new applications. Recently, de Vries and de Rooij proposed a complete framework for applying Graph Kernel (GK) in an adaptive manner to RDF graphs [34]. GK are machine learning methods that have the ability to deal directly with graph data, particularly by computing kernel functions that evaluate similarity between graphs or pieces of graphs [35]. The framework of de Vries and de Rooij is implemented in an open source library named *Mustard* [36]. It enables classifying RDF instances considering their neighborhood in the graph. This neighborhood is encoded within features such as labels of edges or graph substructures such as *walks* (i.e., linear paths) or *subgraphs*. In the work we present here, we reused Mustard and fitted its capability of instance classification to the case of link prediction.

In relation with PGx research, Percha et al. mined the set of RDF statements extracted from text by Coulet et al. with a Random Forest (RF) algorithm and successfully predicted drug–drug interactions [37]. With the aim of predicting pharmacogenes, we experimented as Percha et al. with the RF algorithm in the preliminary stage of this work [38]. First results we obtained with RF are here updated and compared with GK approaches.

### Discovery of pharmacogenes

Hansen et al. proposed a method based on a logistic classifier to generate candidate pharmacogenes, using data from PharmGKB, DrugBank, and protein–protein interactions from InWeb [39]. An issue with this approach is that PharmGKB and DrugBank are manually curated from the literature and are consequently expensive to maintain and update. Garten et al. answered this issue by proposing an automatic method that consider directly (and only) the literature [40]. They improved the results obtained by Hansen et al. by considering gene–drug pairs co-occurring in sentences of the PGx literature. Recently, Funk et al. proposed also to use the biomedical literature, plus GO annotations, to identify pharmacogenes [41]. They obtain a high F-measure and AUC-ROC (0.86 and 0.86), but proposed a coarse-grained classification that is only binary (pharmacogene or not), avoiding any ranking of the candidates.

Semantic web technologies have also been experimented for PGx knowledge discovery. Dumontier and Villanueva-Rosales proposed a knowledge representation of the domain and benefit from reasoning mechanisms to answer sophisticated queries related to depression drugs [42]. Coulet et al. used patient data to instantiate a description logics knowledge base, then extracted association rules from it to identify *gene variant–drug response* associations [43]. More generally, advantages that semantic web technologies may offer to PGx and personalized medicine are listed in [23].

We present here a method that consists in mining a set of diverse linked data sources to help validating uncertain gene–drug relationships. This method can be divided in three steps: *first*, selecting and connecting relevant PGx linked data; *second*, formatting linked data to train and compare two machine learning algorithms (RF and GK); *third*, classify and rank candidate pharmacogenes with these two approaches. The paper is organized as follow: next section presents our methods for preparing, then learning from the linked data; next, *Results* Section presents the evaluation and the use of the two machine learning approaches we considered and brings elements of interpretation; the two last sections discuss our results and conclude on this work.

## Methods

### Data preparation

**Fig. 1:**
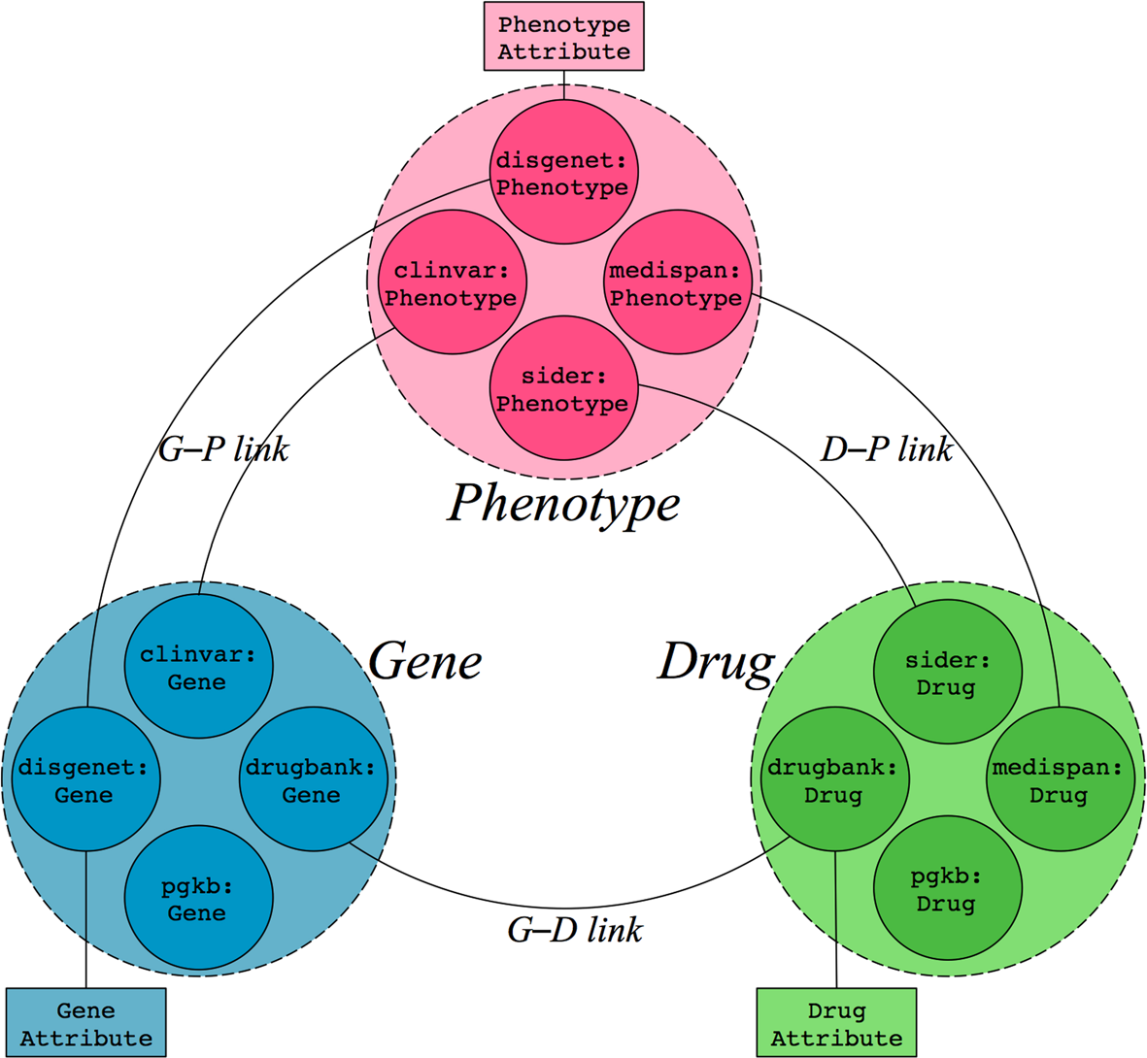
Overview of the type of entities and relationships considered and their origin. Entities are of three distinct types: Gene, Phenotype and Drug. Gene–Phenotype relationships are coming from ClinVar and DisGeNET, Phenotype–Drug relationships from SIDER and Medi-Span, Gene–Drug relationships from DrugBank. In addition, we included gene and drug entities from PharmGKB to enable building the training and test sets. Equivalence mappings are defined between entities of the same type but of different origin. In addition to entity–entity relationships, we consider some attributes that are specific to entities, such as the ATC class of drug that is a drug attribute. Naming of different parts of the data (e.g., G–P links, gene attributes) is used later in the step of formatting of the linked data. The detailed schema of the data is provided Fig. 2

**Data selection** Initial step is to select a set of data that include relevant data about PGx gene–drug relationships. Figure 1 gives a general overview of the type of data we consider for this study: three types of entities, *gene*, *phenotype* and *drug*; and relationships between them, i.e., *gene–phenotype*, *phenotype–drug* and *gene–drug* relationships. We selected data sources manually but oriented our selection to sources providing typed relationships and limited ourselves to two sources per relationship. As a result, we selected ClinVar and DisGeNET for gene–phenotype; SIDER and Medi-Span for phenotype–drug; DrugBank for gene–drug relationships. PharmGKB completes the set of data sources to enable building the training and test sets (see “Learning task, training and test sets” subsection).

**Fig. 2:**
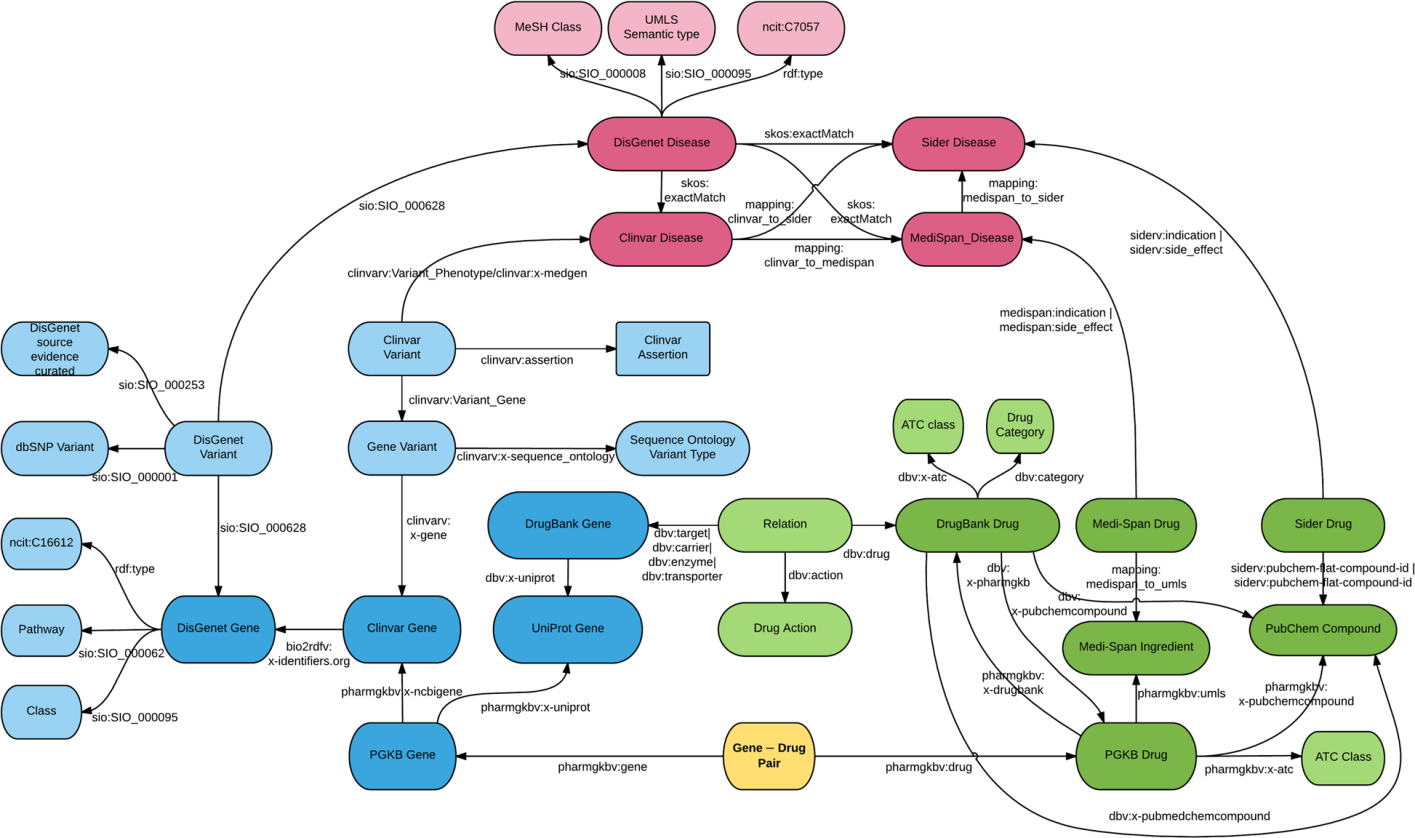
Schema of the pharmacogenomic linked data selected for this study. Entities are related to either Genes, Phenotypes (or Diseases) or Drugs. We artificially enriched the data with an additional type of entity: gene–drug pairs. These entities link exactly one gene and one drug and are the nodes of the graph we classify either as *associated* or *not associated* from a PGx point of view, to valid candidate pharmacogenes. For mapping purposes, we added to our dataset Gene references from UniProt and Drug references from PubChem. Because part of Medi-Span and part of PharmGKB data are protected, we restricted the online access to the data

**Fig. 3:**
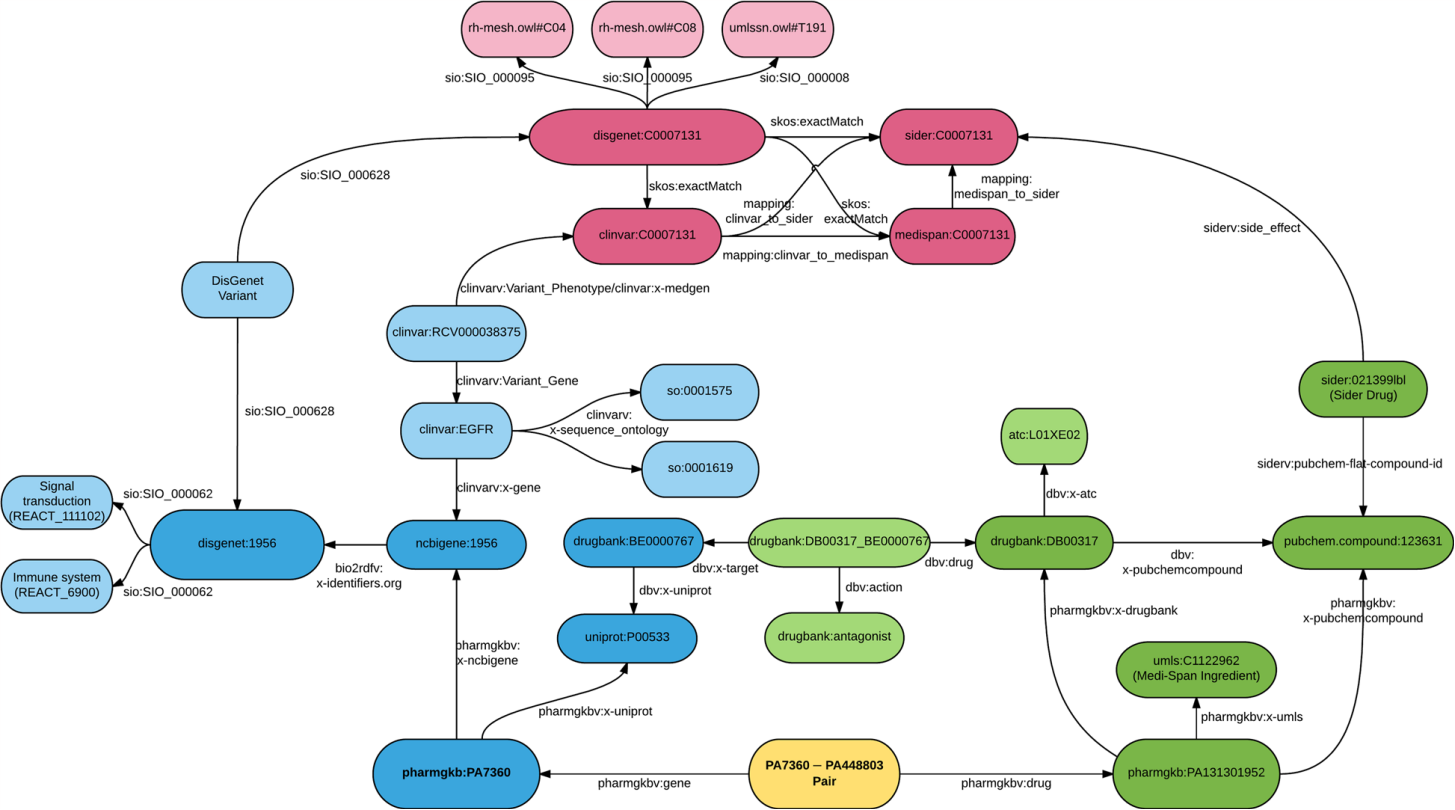
Sample of PGx linked data, surrounding the gene–drug pair between the *EGFR* gene and *Carboplatin*. Entities of same type but of different provenance are mapped to each other. This graph data are used to build features describing this particular gene–drug pair. Additional file 1 provides the SPARQL query that returns this graph when applied to our selection of linked data. Prefixes used in this figure, such as dbv, are fully expanded in the Additional file 1

**Data RDFization** The second step is about turning selected data in a standardized RDF graph, available at https://pgxlod.loria.fr. We benefit from the fact that DisGeNET [44], SIDER [45] and DrugBank [46] are already available online in the form of LOD and reused them. DisGeNET includes data from ClinVar, but because it includes only a part of it, we made our own RDF version of ClinVar following guidelines and scripts of the Bio2RDF project. We completed the Bio2RDF version of PharmGKB locally with gene–drug relationships manually annotated by PharmGKB but not openly distributed [15]. Similarly, we transformed drug indications and side-effects from Medi-Span in the form of RDF triples and loaded them into our SPARQL server. For the management of RDF data, we rely on Blazegraph, a graph database system that provides support for RDF and SPARQL. Medi-Span data, as PharmGKB clinical annotations are protected by a license agreement and can not be redistributed. This explains why we are providing a controlled access to our set of PGx linked data. We propose to open this dataset, on demand, with licensees. Figure 2 presents the detailed schema (i.e., type of entities and relationships) of the linked data we selected and consider for mining. Figure 3 presents an example of data from the PGx linked data, instantiating the schema presented Fig. 2. The SPARQL query returning data presented in Fig. 3 is provided in Additional file 1. Other SPARQL queries, such as the one provided in Additional file 2, may be built by considering the partial data schema presented Fig. 2.

**Mapping definition** To define mappings, we first relied on standard identifiers such as NCBI Gene ID found in DisGeNET and ClinVar URIs and UMLS CUI found in DisGeNET, ClinVar, SIDER and Medi-Span. We defined regular expressions over URIs to isolate identifiers and when two match, we define a mapping. Figure 3 shows two entities, clinvar:1956 and disgenet:1956 that share a unique identifier within different namespaces.

Second, when no standard identifier exists, we relied on services provided by biodb.jp to obtain cross-references between identifiers and, accordingly, define mappings [47]. We implemented a tool named biojp2rdf that transforms the cross-references provided by biodb.jp in RDF [48]. For drugs, we also relied on the API provided by RxNav to obtain mappings between Medi-Span identifiers and UMLS CUIs [49]. We loaded all mapping data into our SPARQL server to enable the resolution of identity between entities of the same type.

### Learning task, training and test sets

**Learning task** Our learning task is a supervised classification of specific nodes from the data graph. Nodes considered for classification represent candidate pairs of one gene and one drug, which are binary classified as either pharmacogenomically associated or not. Each candidate pair is added to the data graph as a new node linked to both the gene and the drug constituting the pair. Figure 3 provides an example of such a pair and its links to its constituents.

**Training set** To constitute our training set we defined two sets of instances: *positives* and *negatives*. Our set of positives is gene–drug pairs annotated as associated according to PharmGKB (version of October 1^*st*^, 2015) and that are annotated with a high level of validation in PharmGKB, i.e., level =1 or 2 [14]. PharmGKB clinical annotations are relating gene variants to drugs, not gene to drugs. We generalized these relationships to manipulate gene–drug relationships. When 2 variants of a same gene are associated with two distinct levels of evidence to the same drug, we conserve only the highest. For instance, the *VKORC1* gene has several variants associated to *warfarin* with level of evidence from 1 to 4. We conserve only that the *VKORC1* gene is associated to warfarin with a level of evidence 1. Accordingly, we generated 91 positive instances.

To constitute our set of negatives, we randomly generated gene–drug pairs from those listed in PharmGKB, but checked to be absent from DGIdb (the Drug Gene Interaction database), which collects gene–drug relationships from various sources [21], including PharmGKB. Two distinct sets of negatives were generated, one of 91 instances to balance exactly with the number of positives and one of 182, to experiment with this unbalance.

**Test set** We considered the 1760 gene–drug pairs insufficiently validated according to PharmGKB, i.e., associated with a level of evidence 3 or 4.

### Learning pharmacogenes with Random Forests

**The Random Forest algorithm** Introduced by Leo Breiman in 2001 [50], Random Forest (RF) is an ensemble method, combining decision trees in order to obtain better results in supervised learning tasks. Let *X* and *Y* compose a training set, where *X*=〈*x*_1_,*x*_2_,...,*x*_*n*_〉 is a vector of feature vectors and *Y* is a vector of classes *Y*=〈*y*_1_,*y*_2_,...,*y*_*n*_〉. A class *y*_*i*_ is accordingly associated with each feature vector *x*_*i*_. In the case of a binary classification, each vector *x*_*i*_ is associated with a value *y*_*i*_ that is either 0 or 1. The method begins by creating several new learning sets, each one being a sample –with replacement– of elements from *X*; described by their classes and a sample of their features. A decision tree is then trained on each learning set to take part in a majority vote, which result is the result of the RF. This approach enables a better accuracy and generalization of the model, and counterbalances the instability of decision trees, a forest being more stable to slight changes in the data.

**Data formatting with RF** Linked data are in the form of graphs, whereas machine learning algorithms such as RF take a feature matrix as an input. Consequently, our PGx linked data requires to be formatted in the form of such a matrix. Each line of a feature matrix represents an instance and each column represents a feature describing the instances. We propose encoding parts of the RDF graph by observable paths that start from the gene and the drug of a gene–drug pair. These paths start from the gene or the drug and potentially reach each others. To contain the size of the matrix, we simplify paths from genes and drugs in path of length 1, hereafter named *G–D link*, *G–P link* or *D–P link*, depending on the entity that are connected. In addition to these links, we encode few *attributes* that qualify drugs, genes themselves and phenotypes that are connected to them through G–P or D–P links. Figure 1 summarizes the elements of the graph we consider in this formatting step. Because several paths may leave a gene or drug, one gene–drug pair may be described in the matrix by several instances. But each instance describes a unique pair. A pair is thus described by the set of instances that represent possible combinations of paths and attributes associated with a pair. As an example, Table 1 shows the matrix obtained when formatting the sample of linked data represented in Fig. 3.

**Table 1:** Example of a feature matrix generated from linked data

ID	Gene attribute	Phenotype	Drug attribute	G-D link	G-P link	D-P link	Class
PA7360-PA131301952	Signal transduction	C0007131	L01XE2	Antagonist	clinvar: so_0001575	sider: indication	1
PA7360-PA131301952	Immune system	C0007131	L01XE2	Antagonist	clinvar: so_0001575	sider: indication	1
PA7360-PA131301952	Signal transduction	C0007131	L01XE2	Antagonist	clinvar: so_0001619	sider: indication	1
PA7360-PA131301952	Immune system	C0007131	L01XE2	Antagonist	clinvar: so_0001519	sider: indication	1

All the instances (e.g., lines) describe the same gene–drug relationships (EGFR–Gefitinib), which is associated in PharmGKB with a high level of evidence (*Class*=1). Figure 3 shows some of the data associated with this relationships in linked data. Values are extracted from the graph and are encoded in various manner. For example, values of phenotypes are UMLS CUI, values of drug attributes are ATC codes

**Multi-instance classification and candidate ranking** With RF classification and GK, a probability distribution value, denoted *p*_*RF*_ or *p*_*GK*_, may be used to evaluate the confidence of the model for classifying a new instance and then rank classified instances. However, the gene–drug pairs that we classify are typical examples of multi-instance objects, also named bag of instances, since they are not represented by a single instance but by several ones. Additional treatment is then required to classify and rank bags of instances. One option, as seen in [51], is to use the max operator, such that *p*_*i*_= max*p*_*ij*_, where *p*_*i*_ is the probability estimate for the bag i, and *p*_*ij*_ the probability estimates of all instances j of the bag i. Another option would be to compute the arithmetic mean $\bar {p}$ of probabilities of instances of the bag [52]. However, one bag of instances can contain at the same time instances classified as positive and instances classified as negative in our case, all with a high *p*_*ij*_. In this case, applying the max or the arithmetic mean operator would lead to false positives. We choose to use a weighted mean to aggregate all the *p*_*ij*_. Let *n*_*i*_ be the number of instances in the bag *B*_*i*_ and Class _*ij*_ the classification decision proposed by the model for the instance *j* of the bag *B*_*i*_.

1 $$ p_{i} = \frac{{\sum\nolimits}_{j = 1}^{n_{i}} {a \times p_{ij}}}{n_{i}}, \text{with}~a =\left\{\begin{array}{ll} -1 & if \quad \text{Class}_{ij} = 0 \\ 1 & if \quad \text{Class}_{ij} = 1 \end{array}\right.  $$

For each bag *B*_*i*_, *p*_*i*_∈ [ −1,1]. If every instance of a bag is associated with a strong confidence for being classified as positive (Class _*ij*_=1), then *p*_*i*_ will be close to 1. In the case of a bag of instances associated with a strong confidence for a negative classification, *p*_*i*_ will be close to -1. *p*_*i*_ close to 0 means that we cannot classify, positively or negatively, the bag with a strong confidence.

### Learning pharmacogenes with graph kernels

**Data formatting for graph kernel** Graph Kernels (GK) present the advantage of handling directly data in the form of graphs. In addition, the Mustard library that we propose using, handles directly RDF graphs as an input, consequently limiting formatting efforts. GK generates the attributes of the instances either as a list of feature vectors or as a kernel matrix. Consequently, we provide to Mustard the PGx linked data that we selected. Mustard is designed to compute RDF node classification, whereas we want computing link prediction. To adapt to Mustard, we enriched the PGx data with artificial entities so we can adapt link prediction to a classification task. Concretely, we add entities to represent gene–drug relationships, each related to a unique gene and drug. In addition, two classes named *associated* and *not associated* are added to the graph and related with pairs of the training set. For example, a positive pair from the training set will be a node in RDF, related to the class named *associated*. Mustard task is to classify test pairs as associated or not. Finally, we needed to invert the direction of some links in our graph since Mustard allows exploring predicates in only one direction, whereas we want kernel functions exploring the full graph without considering the direction of predicates. Enriching the graph with inverse predicates has been considered but this generates many cycles that are indeed considered by some of the graph substructures then computed by the kernel functions (see the next subsection for details).

#### Graph kernels in Mustard

In [34], de Vries et de Rooij proposed a general framework, implemented within the Mustard library, with a list of kernels to generate the features of RDF instances. The framework works as follows: First, the neighborhood of each instance, up to a certain depth, is extracted. Additional file 2 proposes an example of SPARQL query that returns the neighborhood, up to a lenght of 4, of an example drug. Then, predefined substructures are counted within the boundaries of this neighborhood. The attributes of each instance are then the count, for that instance, of the substructures extracted from all neighborhoods.

Mustard includes 3 different types of substructures defined below (see [34] for more details):

- *Bag of labels*: A bag of labels is simply the set of vertex labels in the instance neighborhood.
- *Walks*, up to a certain length: A walk is a set of consecutive edges in the graph.
- *Sub-trees*, up to a certain length: A sub-tree originating at a vertex is the acyclic graph around that vertex.

Note that the description of the substructures does not require the distinction made in RDF graphs between labels of nodes and labels of edges. Indeed the substructure counting algorithms take care of reifying the edges and transforming them to labeled nodes. By varying the size of the neighborhood and precising the type of substructures, one obtain various feature vectors. We list below parameters that can be changed to compute kernels:

- *Exploration depth*: This parameter defines the depth of instance neighborhood from which we extract and count the substructures.
- *Cycles traversal*: During exploration, cycles can be traversed either once, or multiple times. In the latter case, the same nodes in the cycle get repeated, and the obtained neighborhood is a *Tree* (an acyclic graph) rooted at the instance vertex. Otherwise the neighborhood is considered as a *Sub-graph*.
- *Root constraint*: If the neighborhood is a tree, we also consider the constraint in which only substructures that start from the root vertex are counted. This can lead to a faster computation.
- *Substructure depth*: When counting substructures, we can define the maximum length (respectively depth) of the walks (resp. sub-trees).
- *Minimum frequency*: Two of the main differences between RDF graphs and theoretical graphs usually considered in graph mining are that vertices and nodes in RDF have labels, and there is a large number of different labels in the graph. Many labels may be used only once. This leads, if considering labels, to very specific graph patterns, which do not generalize well. To alleviate this problem, Mustard enables imposing a minimum frequency, under which a label is not counted.

The obtained feature vectors are very sparse, which are efficiently computed using matrix dot products, called kernels. Those kernels are adapted to be computed by Support Vector Machine (SVM) algorithm that are classically the learning algorithm on the basis of graph kernels. We used this algorithm in this work.

## Results and interpretation

### Random Forest results

We trained and evaluated our model using the Weka implementation of the RF and a 10-fold cross-validation. First, we performed an information gain analysis, classically used for feature selection, and found out that the feature named *disease attribute* was providing little information to the classifier (*InfoGain*=0.008). We decided to remove this feature from both the training and test sets. Table 3 presents the results of the evaluation on the model trained with unbalanced data, and Table 2 the evaluation on the model trained with balanced data.

**Table 2:** Results of the 10-fold cross-validation of our first RF model

Class	Precision	Recall	F-Measure
1 (positive)	0.944	0.904	0.924
0 (negative)	0.997	0.998	0.998
Weighted Average	0.996	0.996	0.996

This model is trained with 91 positive and 91 negative gene–drug relationships

**Table 3:** Results of the 10-fold cross-validation of our second RF model

Class	Precision	Recall	F-Measure
1 (positive)	0.804	0.998	0.891
0 (negative)	0.994	0.547	0.706
Weighted Average	0.728	0.735	**0** **.** **7** **2** **9**

This model is trained on 91 positive and 182 negative gene–drug relationships

We evaluate two concurrent models trained either with a balanced set of positive and negative pairs (resp. 91 and 91) or an unbalanced set (resp 91 and 182). The model with balanced classes clearly overfits, with a F-measure=0.996 (see Table 2). This is most probably due to the fact that when formatting the data, negative pairs generate much less instances, leading to a large unbalance between positive (108,038) and negative (3197) instances in the feature matrix. A larger set of negative pairs, leading to a more balanced feature matrix, may temper the overfitting. In this case we obtained a F-measure of 0.729 (see Table 3) and a root mean squared error of 0.385.

With this last model, we classified the 1760 pairs (represented by 984,460 instances) of our test set. The top-20 pairs predicated as positive according to our RF model are provided online at [53, 54].

### Graph kernel results

We used the Mustard library as an implementation of a GK framework to perform the two next experiments.

**Evaluating the impact of GK parameters** The purpose of the first experiment is to evaluate the impact of various kernel settings on the RDF graph we consider here. For each kernel a C-SVC (C-Support Vector Classification) support vector machine from the LibSVM library is trained. Each kernel is evaluated with a 10-fold cross-validation, which is repeated 10 times itself with different randomization seeds. Within each fold, SVM parameters are optimized, again using a 10-fold cross-validation.

Table 4 illustrates how the F-measure of our model changes depending on the substructure and the type of neighborhood considered. Table 5 compares F-measures obtained with various neighborhood depth, for a fixed substructure and type of neighborhood. Surprisingly, the F-measure is not strongly impacted by this parameter. We think that this is due to the fact that most important features are in a distance of 4. Table 6 illustrates how F-measures can be impacted by the root constraint. Table 7 shows the impact on the F-measure of imposing a minimum frequency to the type of vertices and edges considered in the mining.

**Table 4:** Comparison of F-measures obtained with various combination of substructure and neighborhood settings. F-measures are averaged over two other parameters: the *depth* of the neighborhood (*d*=4,6,8,10,12,15) and the *length* of the substructures (*l*=4,6,8,10,12,15)

Substructures \ Neighborhood	Graph	Tree
Bag of Labels	0.763	0.781
Walks	0.777	0.797
Sub-trees	0.782	**0.803**

**Table 5:** Comparison of F-measures obtained with different depths of neighborhood

*d=4*	*d=6*	*d=8*	*d=10*	*d=12*	*d=15*
0.807	0.805	0.801	0.803	0.803	0.804

Neighborhood setting=*Tree* and substructures=*Sub-trees*

**Table 6:** Comparison of F-measures obtained with and without the *root constraint*

	w/ Root constraint	w/o Root constraint
Bag of Labels	0.479	0.781
Walks	0.753	0.797
Sub-trees	0.536	**0.803**

F-measures are averaged over the *length* of the substructures (*l*=4,6,8,10,12,15)

**Table 7:** Comparison of F-measures obtained with different *minimum frequency* settings

Min. frequency	2	4	8	16	32
F-measure	0.794	0.780	0.798	0.796	0.774

Neighborhood setting=*Tree* and substructures=*Walks*

**Classifying candidate pharmacogenes** In the second experiment, we trained our model and applied it to our test set to classify candidate pharmacogenes. Regarding the evaluation of the model, a 10-fold cross-validation is done and repeated 10 times with different randomization seeds. Within each fold, we optimize the different kernel settings again using 10-fold cross-validation. The reason for optimizing the kernel settings within an inner cross-validation instead of the selecting the best settings from the previous experiment, is to avoid a model selection bias which can lead to a misleading optimistic performance evaluation as shown in [53, 54].

Table 8 presents the results of the evaluation of the model trained with both balanced and unbalanced data. We report the F-measure for the positive class, the average F-measure and the AUC-ROC. For the best average F-measure, i.e., 0.807, the error rate for a 95% confidence interval is 0.008.

**Table 8:** Results of the 10-fold cross-validation of our Graph Kernel/SVM model

	F-measure	Avg. F-measure	AUC-ROC
*Balanced*	0.770	0.761	0.840
*Unbalanced*	0.746	**0.807**	0.905

Models are trained with 91 positive and 91 negative examples for the *Balanced* model and 91 and 182 for the *Unbalanced*

With the unbalanced model, we classified the 1760 instances of our test set. The top-20 pairs predicated as positive according to our GK model are provided online at [55].

### Result combination

We intersected the two lists of top candidate pairs obtained by RF and GK, to keep only those present in both classification. Then, we sorted the pairs by descending order of *p*_*RF*_. Table 9 presents the 20-top candidates obtained by this method. We notice that with this ranking both *p*_*RF*_ and *p*_*GK*_ are closed to 1. A PGx expert (NCN) examined the 20-top candidates within a manual literature study to evaluate their relevance and estimate their interest for further investigation. Results of this examination are reported in the next subsection.

**Table 9:** 20-Top candidates of gene–drug pairs predicted from our PGx linked data

Rank	Gene	Drug	*p*_*RF*_	*p*_*GK*_
1	*MAP3K1*	Carboplatin	0.993	0.991
2	*EGFR*	Erlotinib	0.992	0.980
3	*EGFR*	Fluorouracil	0.989	0.966
4	*FCER1G*	Aspirin	0.988	0.993
5	*MAP3K1*	Erlotinib	0.988	0.830
6	*DSCAM*	Carboplatin	0.979	0.974
7	*CHIA*	Aspirin	0.979	0.911
8	*GP6*	Aspirin	0.979	0.976
9	*ACE*	Sidenafil	0.979	0.911
10	*TPMT*	Cyclophosphamide	0.975	0.994
11	*CYP2B6*	Nicotine	0.973	0.912
12	*PTGER3*	Aspirin	0.967	0.965
13	*NTRK1*	Aspirin	0.967	0.992
14	*EXO1*	Fluorouracil	0.966	0.694
15	*ERBB2*	Trastuzumab	0.964	0.998
16	*CYP2B6*	Olanzapine	0.964	0.965
17	*HLA-DQ1*	Azathioprine	0.963	0.931
18	*HMGCR*	Simvastatin	0.963	0.996
19	*CYBA*	Simvastatin	0.961	0.973
20	*HLA-DRB1*	Mercaptopurine	0.961	0.966

### Interpretation

Among the top-20 candidates obtained with both predictions models (Table 9), unreleased gene–drug pairs which should be further investigated were combined to extensively-studied candidates, not surprinsingly mainly in cancerology.

For instance, it is widely known that aberrant epidermal growth factor receptor (EGFR) signaling lead to various oncogenic phenotypes [56] and previous PGx invetigations have shown that the *EGFR* gene mutation status was associated with EGFR-targeted agents efficacy such as Erlotinib’s (rank 2) in the case of non-small cell lung cancer (NSCLC) [56, 57]. In addition to its single agent activity, it has also been shown that this tyrosine kinase inhibitor acts in synergy with standard chemotherapy such as Fluorouracil in various cancer patients [58, 59] and we were able to pair Fluorouracil with the *EGFR* gene as well (rank 3).

Conversely, the *MAP3K1* gene’s association with Carboplatin (rank 1) seemed novel and yet, in a genome-wide association study on advanced NSCLC patients treated with this antineoplastic chemotherapy drug, a single nucleotide polymorphism in the *DSCAM* gene has been identified as a prognostic biomarker candidate [60]. This supports our drug-gene pair in rank 6 and gives insights on possible *MAP3K1* ×*DSCAM* synergy that should be further investigated.

Those various outputs (confirming bibliography or unreleased) show that our approach could be of value in *1)* strengthening PGx knowledge and facilitate its translation in practice and *2)* leading to novel investigations in order to better identify the complex synergies in action.

## Discussion

We considered first the RF algorithm because it has been successively applied for the prediction of drug–drug interactions from a set of RDF statements [37, 48]. The availability of the Mustard library and its results in term of node classification motivates us to compare RF with GK. One drawback of the Graph Kernel method is that it is not always possible to know which part of the graph data have the biggest contribution to classification since a graphs is classified by similarity. This may motivate the investigation of other subgraph mining methods that may be more informative on the weights of substructures in the classification. One may consider techniques such as gBoost [61] that progressively collects informative patterns, or gSpan [62] that enumerates frequent subgraph used as features for classification.

RF algorithm performs correctly (F-m =0.73) in the frame of our case study, but presents several limitations. First, RF is limited by our usage of a multi-instance representation of data, i.e., data about one gene–drug pair is represented in the feature matrix by several lines [52]. Because our dataset contains much more data about positive examples than data about negatives, it results that if we balance the number of positive and negative examples in the training set (respectively 91 and 91), the actual number of lines (i.e., instances) in the matrix describing positive examples is much larger than for negatives (respectively 108,038 and 3,197). However, our experiments showed that initial selection of negative examples, as well as keeping a certain unbalance, is important. This large unbalance lead the RF to an overfitting, i.e., an instance to classify will most probably be similar to one of the numerous descriptions of positive examples and be classified as positive. We overcome this drawback by doubling the number of negative examples in our training set. This unbalancing of examples (respectively 91 positives and 182 negatives) resulted in a reduction of the unbalance in the feature matrix (respectively 108,038 and 57,885). Here we can note that the second set of negative example is described by much more instances than the first one, what let us see that our random pick of negative examples in the large set of gene-drug relationships not referenced by DGIdb impacts to some extent the results of our approach.

Another limitation of RF is that it requires a formatting of the graph data and then to select a set of features. We achieved this selection manually to retain 6 features, and then apply a standard feature selection method (Information Gain) that enabled us to filter one useless feature (disease attribute such as the MeSH class of the disease).

Our experiment shows that GK achieves globally better than RF, and that the Mustard library offers many facilities to mine RDF data. In addition, it provides us several insights on the features that are more relevant to consider when mining our PGx linked data. For example, it seems that considering only the close neighborhood (*d*=4) of instances to classify is sufficient in our case. Also, in the case of a constrained graph, as the one we designed, considering substructure in the neighborhood of instance may not be of primary importance. This may be associated to the fact that we limited the size and connectivity of the data graph, and consequently knowing solely the label of a set of edges and of vertices may enable to reconstruct a path or a subgraph in the neighborhood of an instance.

Our selection of data sources may be discussed, particularly because some of those are not open. Of course adding new sources would be of interest. Indeed, our choice for the linked data framework is motivated by the fact that we want to ease the addition/removal of data sources for enabling the selection of best features out of many sources without considering if they are open or not. In regards with the results from previous works [40, 41], we think that sources of triples extracted from the literature would be particularly valuable, such as those extracted in [25]. Because GK considers data directly in the form of a graph, one could want to mine directly LOD resources, without particular selection. However, the large number of available data in the LOD, including many non-informative metadata, makes this still challenging. We decided to use multiple data sources, with various license agreement. Further work could evaluate the impact of adding/removing data sources, then offering the opportunity to compare the importance of open data vs. not open data.

A limitation to our approach is related to the field of PGx itself since only few (91) gene–drug relationships have a high level of evidence according to PharmGKB, making our training set relatively small. One way of enlarging the size of the training set would be to consider *gene variant–drug* relationships, instead of gene–drug, that have two advantages: being more numerous, but also rendering more precisely the state of the art of PGx knowledge. Indeed, a gene may host two (or more) variants, one that impacts drug response and one that does not.

Two biases are to consider when interpreting the results. First, negative examples are gene–drug relationships not listed in DGIdb, which includes known and predicted candidates from many databases. Consequently, negatives are likely not to be related, instead of not being related, but to our knowledge no existing resource lists negative gene–drug relationships. Second, tested examples are likely to be related since they are listed in PharmGKB with a low level of confidence. However, our goal is prioritize these candidates, instead of detecting negatives from those.

## Conclusion

This article is a proposal to help validating candidate pharmacogenes by learning from PGx linked data. More precisely, we selected and interconnected data relevant to the PGx domain in the form of a large RDF graph. Then, we formatted these data to train and compare a RF and a GK classifier. These two classifiers were evaluated and used to identify and rank candidate pharmacogenes. GK achieves a F-measure of 0.81, whereas RF reaches 0.73. Top candidate pharmacogenes pointed out by our approach are provided and interpretated in this article. Top candidates that are not already extensively studied will be further investigated by PGx experts. Results we obtained with the GK library named Mustard are particularly promising both for our application domain, i.e., validating pharmacogenes, and more broadly for the mining of biomedical linked data.

## Additional files

Additional file 1 SPARQL query example 1. This text file contains the SPARQL query we apply on our PGx linked data to obtain the data graph represented in Fig. 3. This query includes the definition of prefixes mentioned in Figs. 2 and 3. This query takes about 30 s on our https://pgxlod.loria.fr server. (TXT 2 kb)

Additional file 2 SPARQL query example 2. This text file contains an example of SPARQL query that enable to explore the vicinity of an entity. This particular query returns the RDF graph surrounding, within a lenght of 4, the node pharmgkb:PA451906 that represents the *warfarin*, an anticoagulant drug. (TXT 392 bytes)

## Abbreviations

AUC-ROC: Area under the receiver operating characteristic curve’
GK: Graph kernel
LOD: Linked open data
PGx: Pharmacogenomics
RDF: Resource description framework
RF: Random forest
SPARQL: SPARQL protocol and RDF query language
SVM: Support vector machine
URI: Uniform resource identifier
